# Demarcating the membrane damage for the extraction of functional mitochondria

**DOI:** 10.1038/s41378-018-0037-y

**Published:** 2018-12-31

**Authors:** Md Habibur Rahman, Qinru Xiao, Shirui Zhao, Fuyang Qu, Chen Chang, An-Chi Wei, Yi-Ping Ho

**Affiliations:** 1 Department of Biomedical Engineering, The Chinese University of Hong Kong, Shatin, New Territories, Hong Kong SAR, China; 2 Shun Hing Institute of Advanced Engineering, The Chinese University of Hong Kong, Shatin, New Territories, Hong Kong SAR, China; 3 Graduate Institute of Biomedical Electronics and Bioinformatics, National Taiwan University,

## Abstract

Defective mitochondria have been linked to several critical human diseases such as neurodegenerative disorders, cancers and cardiovascular disease. However, the detailed characterization of mitochondria has remained relatively unexplored, largely due to the lack of effective extraction methods that may sufficiently retain the functionality of mitochondria, particularly when limited amount of sample is considered. In this study, we explore the possibility of modulating hydrodynamic stress through a cross-junction geometry at microscale to selectively disrupt the cellular membrane while mitochondrial membrane is secured. The operational conditions are empirically optimized to effectively shred the cell membranes while keeping mitochondria intact for the model mammalian cell lines, namely human embryonic kidney cells, mouse muscle cells and neuroblastoma cells. Unsurprisingly, the disruption of cell membranes with higher elastic moduli (neuroblastoma) requires elevated stress. This study also presents a comparative analysis of total protein yield and concentrations of extracted functional mitochondria with two commercially available mitochondria extraction approaches, the Dounce Homogenizer and the Qproteome^®^ Mitochondria Isolation Kit, in a range of cell concentrations. Our findings show that the proposed “microscale cell shredder” yields at least 40% more functional mitochondria than the two other approaches and is able to preserve the morphological integrity of extracted mitochondria, particularly at low cell concentrations (5–20 × 10^4^ cells/mL). Characterized by its capability of rapidly processing a limited quantity of samples (200 μL), demarcating the membrane damage through the proposed microscale cell shredder represents a novel strategy to extract subcellular organelles from clinical samples.

## Microfluidics: extracting mitochondria

A microfluidic device enables the shearing of cell membranes without causing substantial damage to mitochondria. Understanding the mechanisms of mitochondria is facilitated by isolating them from their host cell, so that they can be studied without influence from other organelles. Shearing the cell membrane to release the mitochondria is a common approach, but can also result in damage to the mitochondria. Now, a team led by Yi Ping Ho from The Chinese University of Hong Kong reports a “microscale cell shredder” based on a cross-junction microchannel device, in which the cell suspension and buffer are introduced into opposite ends, with the resulting stretch of cell membrane, releasing the mitochondria. The “microscale cell shredder” achieves a 40% increase in extracted functional mitochondria, as compared to existing approaches.

## Introduction

Mitochondria, known as the power house of cells, are prominently responsible for the energy production through producing ATP by respiration. Besides the bioenergetic functions, mitochondria are critically involved in metabolic tasks regulating the physiological responses of cells such as cell signaling *via* reactive oxygen species^1,2^, cell differentiation and death^3^. Mitochondrial dysfunction, typically referred to as malfunction of mitochondria for the cellular adaptations to environmental alternations^4^, has been recently found associated with major human diseases including cancers^5^, neurodegenerative disorders^6^, premature aging^7^ and several cardiovascular diseases^8^. Thus, analyses of the contents and functions of mitochondria have become an important undertaking to further elucidate the role of mitochondrial defects in disease development. An assessment of mitochondria in the cells may illuminate their cytosolic functions when surrounded by cytoskeleton and other subcellular organelles^9^. However, mitochondria grow in the form of complex reticular network in living cells and undergo continuous structural alternations^10^, which complicates the characterization of mitochondria in cells. Therefore, to understand the mitochondrial intrinsic properties without the interference of other subcellular organelles, in vitro analysis of mitochondria remains the mainstream^11^.

The foremost task of in vitro mitochondrial analysis is the extraction of mitochondria, where the cell membrane is either disrupted physically or lysed chemically to release the cellular contents, followed by the fractionation of mitochondria from other subcellular organelles by density gradient centrifugation or immunocapture^12^. As implied by the procedures, an important requirement for the mitochondrial extraction is to disrupt the cellular membrane while maintaining the integrity and functionality of mitochondria. Chemical-based cell lysis largely relies on enzymatic degradation of cellular membrane by membrane poring enzymes such as Streptolysin-O^13^. While the chemical lysis may effectively damage the cell membrane, the mitochondrial membrane may also be impaired under the exposure of membrane digesting enzymes^14^. Physical rupture of cellular membranes is typically conducted by nitrogen cavitation, sonication or mechanical homogenization. Nitrogen cavitation generates bubbles by releasing high pressurized liquid nitrogen, which tears up the cell membrane and releases the subcellular components^15,16^. However, the extracted subcellular organelles become fragile after the process of nitrogen cavitation. Further, the effectiveness of nitrogen cavitation depends largely on the cell types as the membrane properties of different cells and subcellular organelles (especially mitochondria) may vary significantly^17^. Sonication employs ultrasonic waves to mechanically break the cell apart and release the cellular contents, a process typically referred to as sonoporation. Though sonoporation is effective in disrupting the cellular membrane, the high energy introduced in the process may generate heat and subsequently alter the function of extracted organelles, or more problematically, nonspecifically disrupt the mitochondrial membranes^18^. Both nitrogen cavitation and sonoporation are time-consuming procedures and suffer from unfaithful optimization against different cell types of different mechanical properties. Overall, quantitative assessments are lacking for cell membrane damage in response to different operational parameters. In general, chemical lysis, nitrogen cavitation, and sonoporation are not preferred for mitochondrial extraction when mitochondrial integrity and functionality are prioritized.

Considering the versatility, the most widely used method for mitochondria extraction is perhaps the homogenization^19^, where the cell lysates are prepared by mechanically shearing the cell membrane using a Teflon-glass apparatus such as Dounce Homogenizer. The cells are typically placed in a mortar and sheared by a well fitted pestle. The level of shear is collectively determined by the clearance between the pestle and the mortar, as well as the number of strokes and the grinding speed, therefore applicable to extract mitochondria from cells of different membrane stiffness. However, existing geometrical constraints of the homogenizers have limited their applications to relatively large volume (typically 1,000 μL minimally) and high sample concentrations (more than 10^6^ cells/mL)^20^. Further, the outer membrane integrity of extracted mitochondria has been reduced to 40% when cells are disrupted by the homogenizer^18^. Although the easy accessibility has made Dounce Homogenization a standard procedure for mitochondrial extraction, the poor reproducibility when handling low sample volume and concentration has significantly hindered the use of Dounce Homogenizer for clinical samples. Though there are other automated versions of homogenizers such as rotor-stator homogenizers and high-pressure homogenizers available in the market, the systems are either expensive or remain unrealistic for handling low concentration of clinical samples. Last but not the least, the semi-quantitative optimization based on the number of strokes and the grinding speed is plausibly operator dependent and therefore difficult to be standardized.

Continuous development in microfluidics has proven the advantages of miniaturizing some lab procedures on-chip with significant reduction of sample consumption and improved reproducibility^21^. When cell homogenization is considered, several innovations have been proposed to fragment cells^22,23^ and to extract intracellular components, such as the genetic contents and proteins. For instance, nanoscale barbs^24^ and ultra-thin nanoblades^25,26^ have been introduced to disrupt the cells and subsequently extract the proteins. The force employed by the nanoscale barbs or sharp nanoblades are effective for extraction of nucleotides^27^, but the brutal force may endanger the integrity of the subcellular components. External forces such as electrical, magnetic fields and acoustic waves have also been introduced to disrupt cellular membrane, however, long-term exposure to the external forces may have an adverse effect on the functionality of the subcellular organelles such as mitochondria^28,29^. Hydrodynamic stress produced inside microfluidic chip has been utilized to transiently open cellular or nuclear membranes to encourage intracellular gene delivery^30,31^, as well as to significantly disrupt the cellular membrane for DNA/RNA or protein extraction^32^. However, the potential of hydrodynamic stress for the extraction of subcellular organelles is rarely investigated. An initial promise of using hydrodynamic stress to extract mitochondria from small amount of sample (250 µL) has been shown by a cascaded microchannel, where the width of the channel is gradually diminished from 20 µm to 5 µm^33^. The cellular membrane has been significantly stressed by the cascade microchannels and the yield of mitochondrial extraction has shown improved. While the mitochondrial membrane is most likely to be damaged by the high level of stress provided by the cascade microchannels, the integrity and functionality of extracted mitochondria have not been investigated at all. Furthermore, the constrained geometries of the cascaded channel may promote nonspecific binding between the subcellular components and the channel surfaces, resulting clogging inside the microchannel^27^.

**Fig. 1:**
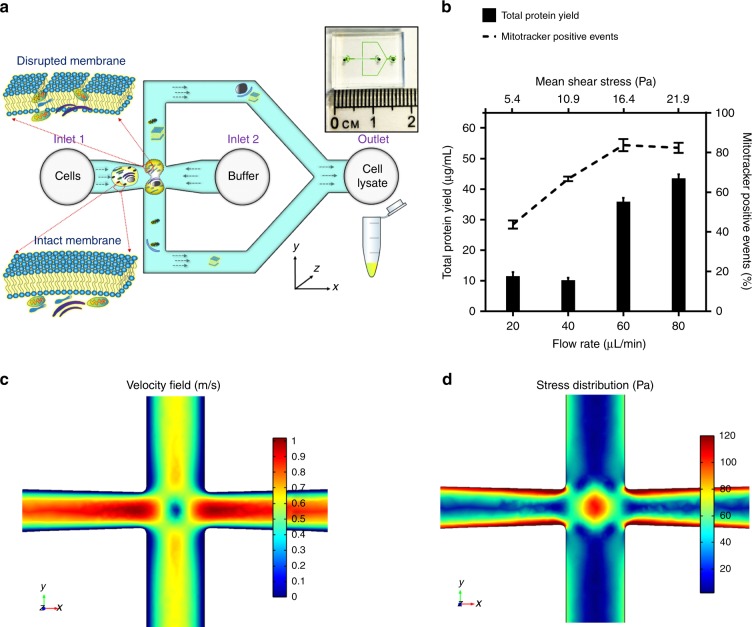
Working principle of the proposed microscale cell shredder. **a** Cells are introduced into the cross-junction of the microchannel. The stress applied on the cell is optimized to disrupt the cell membrane and release subcellular components, while maintaining the integrity of mitochondria. The overview of the microfluidics chip is shown in the inset. **b** The applied mean stress, modulated by controlling the volumetric flow rate for a given channel geometry, has been optimized by the maximal protein yield (an indication of quantity of the extracted subcellular contents) and the maximal mitotracker positive events (a hallmark of functional mitochondria). Results were obtained by shredding HEK293 cells (10^6^ cells/mL) by a range of shear stress and plotted as mean ± SD (*n* = 3 independent experiments). A finite element simulation model was established by COMSOL Multiphysics® to illustrate the fluidic flow at the cross-junction. Give a volumetric flow rate at 60 μl/min, **c** illustrates the velocity profile and the stagnation point at the centre (where the flow velocity is zero), and **d** illustrates the stress distribution and the extensional flow fields around the stagnation point, which contributes significantly to the cell deformation and disruption

Inspired by a recent finding that the shear-induced cellular membrane stretching may physically differentiate the phenotypes of cells by their deformability^34^, this study revisits the possibility of modulating the hydrodynamic stress to selectively disrupt the cellular membrane while maintaining the integrity of mitochondria. Based on previous findings^35^ and our observations, the membranes of mammalian cells are permanently deformed when subjected to a mean shear stress of around 2–10 Pa under laminar flow. Given the elastic modulus of mitochondrial membrane is higher than that of the cellular membrane^36–38^, it shall be possible to observe a threshold stress that may effectively disrupt the cellular membrane while keeping the integrity of mitochondrial membrane. To validate this postulation, we design a cross-junction microchannel, as illustrated in Fig. 1a, where the two streams of reagents, the cell suspension and buffer, are introduced into the cross-junction microchannel from opposite directions. At the cross-junction, the fluid momentum produces high velocity gradient surrounding the stagnation point and the extensional flow fields, which have been shown effective to deform or damage the membranes of mammalian cells^39,40^. The magnitude of stress, determined by the channel geometry and volumetric flow rate^41^, has been empirically demarcated to shred the cellular membrane but to preserve the integrity of mitochondrial membrane. The intactness of cellular and mitochondrial membranes as well as the functionality of mitochondria have been investigated when the cells are exposed to different stress.

We have also explored the potentiality of this “microscale cell shredder” for mitochondrial extraction by comparing with two commercially available approaches, the Dounce Homogenization and the Qproteome^®^ Mitochondria Isolation Kit^11^ using three model cell lines of different membrane stiffness, namely the human embryonic kidney cells (HEK293), mouse muscle cells (C2C12) and neuroblastoma cells (SH-SY5Y). Aside from the obvious advantages of low sample consumption and batch processing inherited by the microfluidics, the microscale cell shredder is observed to yield a greater quantity of extracted mitochondria and maintain higher mitochondrial membrane integrity compared to the two commercially available approaches, particularly at low cell concentrations. To the best of our knowledge, this is the first demonstration of extracting intact and functional mitochondria *via* the microscale hydrodynamic stress. The capability of processing small amount of samples with low concentration is considered particularly favorable for the clinical investigations of mitochondria relevant disorders.

## Results

### Hydrodynamic stress induced disruption of cellular membrane and subsequent extraction of mitochondria

The volumetric flow rates operated in this study were empirically determined against the three cell types. The optimization was established based on concurrent characterizations of the quantity of the extracted subcellular contents and the percentage of functional mitochondria determined by the total protein yield and the mitotracker staining in the extracted subcellular components, respectively. As exemplified in Fig. 1b, at a fixed cell concentration (10^6^ HEK293 cells/mL), the level of cell disruption increased by the flow rate, or the amount of mean stress introduced (Fig. 1b, black bars), as measured by the total protein yield. The extensional stress exerted by the designed cross-junction was evaluated by a simulation model established by COMSOL Multiphysics^®^ (detailed in the Supplementary Text 1). Figure 1c plotted the velocity gradient, whereas Fig. 1d showed the stress distribution surrounding the stagnation point at a flow rate of 60 µL/min. Given the flow rates (20–100 μL/min) operated in this study, the mean shear stress and extensional stress provided at the cross-junction were in a range of 5–20 Pa and 28–180 Pa, respectively, against the three cell types tested. In accordance with the previously reported values for HEK293^35^, significant percentage of cell disruption was observed at the mean shear stress of 10 Pa and the maximal cell disruption occurred at the mean shear stress of 20 Pa (Fig. 1b). Similar phenomena were also observed for C2C12, which has coherent elastic modulus as HEK293 cells^42^. Upon the cell membrane is disrupted, subcellular organelles such as mitochondria can be released, as indicated by the increased mitochondrial positive signals under increased mean shear stress (Fig. 1b). As expected, the slight decrease of mitochondrial membrane potential positive signals suggests that the mitochondrial membrane may also be endangered upon elevated mean shear stress (>16.4 Pa). Therefore, a volume flow rate of 60 µL/min which is equivalent to 16.4 Pa of mean shear stress was employed for the subsequent investigations for HEK293 and C2C12 cell lines.

### Cell disruption and protein extraction efficiency altered by the cell concentrations

**Fig. 2:**
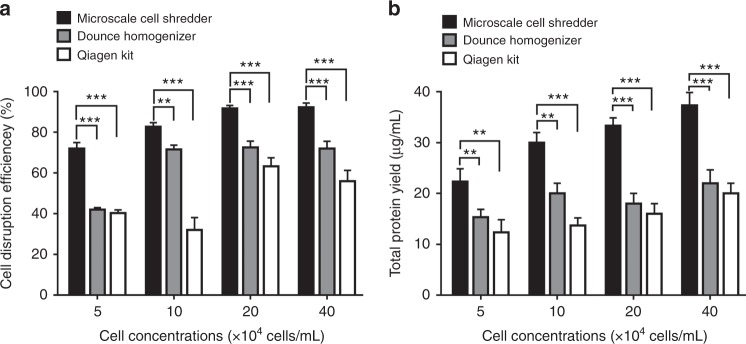
Cell disruption and protein extraction efficiency using the microscale cell shredder, the Dounce Homogenizer and Qiagen Mitochondria Isolation Kit. **a** Cell disruption efficiency, determined by the fraction of disrupted cells against total number of intact cells, was quantified at different cell concentrations. **b** After the centrifugation steps, total protein yield was determined accordingly. These experiments were conducted using HEK293 cells. Results were plotted as mean ± SD (*n* = 3 independent experiments, **P < 0.01, ***P < 0.001)

To further understand the capacity of the miniaturized cell shredder for mitochondria extraction, we compared the performance with two commercially available approaches, the Dounce Homogenizer and Qproteome^®^ Mitochondria Isolation Kit, Qiagen (Qiagen Kit), as the standards of disrupting cells in the mechanical and chemical formats, respectively. The number of strokes for Dounce Homogenizer has been optimized in terms of the total protein yield and percentage of mitochondrial membrane potential prior to the comparison (Supplementary Figure S1), whereas the Qiagen Kit mitochondria extraction procedures were conducted following the manufacture’s protocols. A range of cell concentrations (HEK293) and sample volume were investigated while Fig. 2 plotted the results obtained when small sample volume (200 μL) and low cell concentrations (5–40 × 10^4^ cells/mL) were processed. As indicated in both the percentage of cell disruption (Fig. 2a, fraction of disrupted cells versus total cells) and total protein yield (Fig. 2b, amount of extracted subcellular contents after centrifugation) the microscale cell shredder excelled the performance of Dounce Homogenizer and Qiagen Kit. It is of note that the efficiency of cell disruption drops consistently for all methods when the cell concentration is at the lower bound (5 × 10^4^ cells/mL).

### Quantity and quality of the extracted mitochondria

**Fig. 3:**
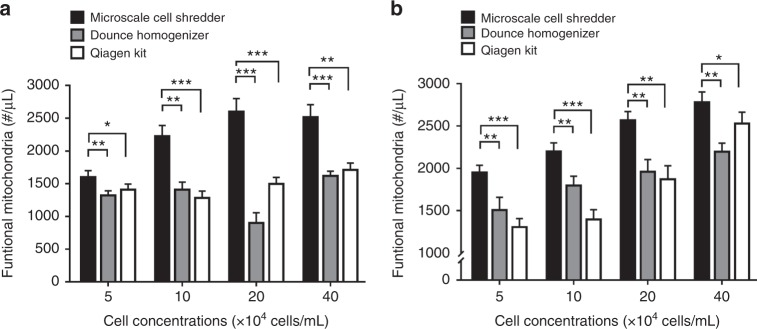
Numbers of extracted mitochondria. Concentrations of functional mitochondria (number per unit volume) in the extracted sample were measured by mitotracker staining, extracted by the three approaches. **a** HEK293 cells, and **b** C2C12 cells. Results were plotted as mean ± SD (*n* = 3 independent experiments, *P < 0.05, **P < 0.01, ***P < 0.001)

Though the membrane disruption efficiency has been improved by shredding the cells at the cross-junction, the microscale cell shredder does not necessarily ameliorate the quality of extracted mitochondria. We therefore investigated the number of functional mitochondria after separating the subcellular organelles. A positive stain of mitotracker, accumulate based upon the membrane potential of mitochondria, was used to quantify the amount of “functional” mitochondria^43^. As observed in Fig. 3, the microscale cell shredder is able to extract around 40% more functional mitochondria compared to Dounce Homogenizer and Qiagen Kit at the cell concentration of 20 × 10^4^ cells/mL for both HEK293 (Fig. 3a) and C2C12 (Fig. 3b). For other concentrations, the microscale shredder also functioned consistently better than the two commercially available approaches.

**Fig. 4:**
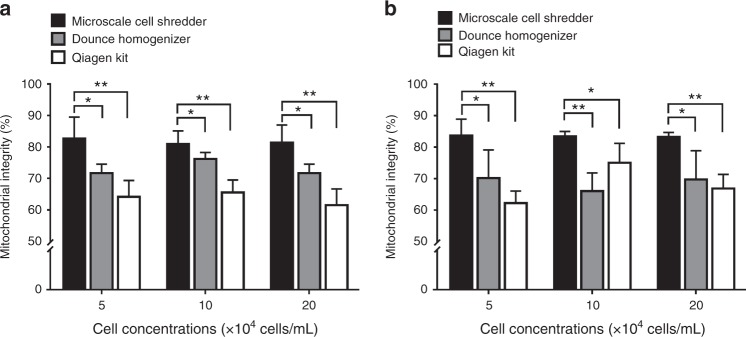
Membrane integrity of the extracted mitochondria. Mitochondrial integrity was measured by the reverse correlation with the released citrate synthase for **a** HEK 293 cells **b** C2C12 cells. Results were plotted as mean ± SD (n = 3 independent experiments, *P < 0.05, **P < 0.01)

Serving as a direct measurement to further evaluate whether the extracted mitochondria remains integrated, the Citrate Synthase (CS) assay was used to determine the citrate synthase activity from the damaged mitochondria^18,44^. The mitochondrial integrity was determined by the difference between total activity (Supplementary Figure S2) and latent activity of the enzyme^18^ as described in the Section of Experimental Methods. As shown in Fig. 4, the microscale cell shredder retained higher integrity of mitochondria compared to the other two approaches particularly when low cell concentrations (5–20 × 10^4^ cells/mL) were involved. The results also corroborated with the enhanced concentrations of Mitotracker red positive signals at the low cell concentrations as observed in Fig. 3 previously.

### Stiffness of cell membrane matters

**Fig. 5:**
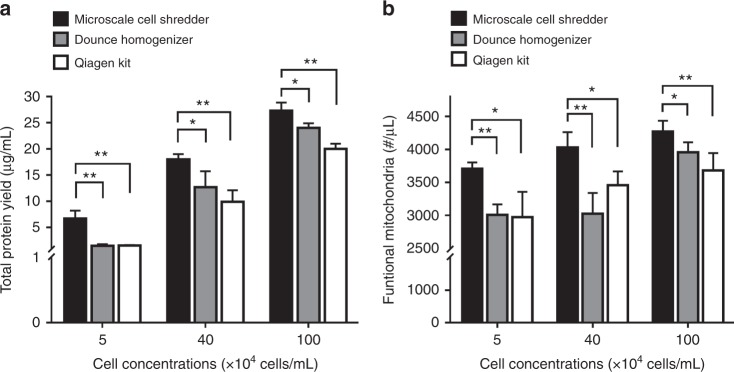
Disruption of neuroblastoma cells (SH-SY5Y) and the subsequent mitochondrial extraction. **a** Total protein yield and **b** concentrations of functional mitochondria obtained from the three extraction methods. Results were plotted as mean ± SD (*n* = 3 independent experiments, *P < 0.05, **P < 0.01)

The fidelity of the proposed concept was validated by disrupting neuroblastoma cells (SH-SY5Y), as the SH-SY5Y cell membrane has higher elastic modulus than HEK293 and C2C12 cells membrane^42,45^. The volumetric flow rate in the setting of microscale cell shredder (Supplementary Figure S3a) and the stroke numbers for Dounce Homogenizer (Supplementary Figure S3b) were again optimized according to the cell disruption efficiency and the positive signal percentage of mitochondrial membrane potential in a similar manner as previously described. Results shown in Fig. 5 demonstrated the same trend for SH-SY5Y cells that the microscale cell shredder yielded significantly greater amount of protein (Fig. 5a) and higher concentrations of functional mitochondria (Fig. 5b) compared to the Dounce Homogenizer and Qiagen Kit, particularly at the low cell concentration (5 × 10^4^ cells/mL).

### Effect of the buffer hypotonicity on cell disruption and mitochondrial extraction efficiency

**Fig. 6:**
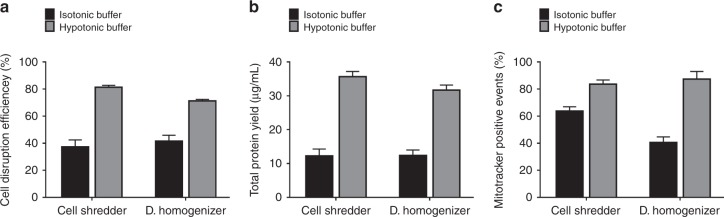
Effect of the buffer hypertonicity. **a** Cell disruption efficiency, **b** total protein yield, and **c** percentage of functional mitochondria measured when the isotonic buffer and hypotonic buffer were used for mitochondrial extraction using the microscale cell shredder and the Dounce Homogenizer. Experiments were conducted by disrupting HEK293 cells of 10^6^ cells/mL and results were plotted as mean ± SD (*n* = 3 independent experiments)

Previously discussed results were conducted using the hypotonic buffer, following a widely adopted protocol to elicit hypotonicity-induced membrane swelling and consequently reduce the membrane stiffness to allow efficient membrane disruption^19^. To address whether the use of hypotonic buffer might disintegrate the mitochondrial membrane, this study continued to investigate the possibility to extract mitochondria using isotonic buffers. The volumetric flow rate for microscale cell shredder and the stroke numbers for Dounce Homogenizer were optimized empirically in terms of cell disruption efficiency, total protein yield and mitochondrial membrane potential for HEK293 cells in an isotonic buffer (phosphate-buffered saline, PBS). Figure 6 shows the results obtained at the same volumetric flow rate (60 µL/min) and the same stroke numbers (10 strokes) for the microscale shredder and Dounce Homogenizer. For the cell concentration tested (10^6^ HEK293 cells/mL), more effective cell disruption (close to double for both cases of microscale cell shredder and Dounce Homogenizer) and mitochondrial membrane potential positive percentage (20% more for the microscale cell shredder and 45% more for the Dounce Homogenizer) were observed when the hypotonic buffer was used. However, it was possible to obtain a similar level of extraction (*i.e*. 75% of mitotracker positive events) using the isotonic buffer (Supplementary Figure S4), though the required volumetric flow rate was higher (80 µL/min versus 60 µL/min for the hypotonic buffer).

## Discussion

The elastic modulus of mitochondrial membrane has been previously characterized higher than that of the cellular membrane^36–38^. This study has investigated the possibility to selectively disrupt the cell membrane but retain the integrity of mitochondrial membrane by modulating the stress inside a microfluidic cross-junction. The mean shear stress employed in this study (~15 Pa) is at the same order of magnitude with previous observations where a shear stress at around 10 Pa under a continuous flow may permanently disrupt the HEK293 cell membrane^35^. Perhaps more reflecting the mechanical damage of cells within a cross-junction, Bae *et al*. has reported a critical extensional stress of around 250 Pa to significantly damage the chinese hamster ovary (CHO) cells^40^. Given that HEK293 cells are ~30% softer than CHO cells based on the Young’s modulus measured by electro-deformation^46^, the critical extensional stress at approximately 105 Pa appears reasonably effective to cause selective damage to the HEK293 cell membrane (Supplementary Figure S5 shows cell deformation at the extensional flow fields) without disintegrating mitochondrial membrane. Following similar rationale, the demarcated extensonal stress to extract functional mitochondria from C2C12 (similar stiffness to HEK293) and SH-SY5Y (~40% stiffer than HEK293^42,45^) cells are estimated to be 105 Pa and 180 Pa, respectively. Interestingly, almost twice amounts of functional mitochondria have been observed from the SH-SY5Y extraction (3,700 ± 100/µL) compared to HEK293 (1,600 ± 100/µL) and C2C12 (1,950 ± 50/µL) extractions for the same cell concentrations (5 × 10^4^ cells/mL). Neuron cells are known to have extraordinarily high metabolism rate^47^ which requires large amount of ATP synthesis and potentially a recruitment of extended amounts of mitochondria in the cytosol^48^. However, the quantitative assessment of how metabolism modulates the numbers of mitochondria in cells hinges on practical techniques to study per-cell organelle numbers^49^. The proposed extraction methodology has shown great potential to effectively retain the mitochondrial functionality and is expected to allow an improved estimation of organelle numbers for the *in vitro* analysis of mitochondria.

Although a necking section is included in the channel design to ensure that the cells are focused laterally to the center of flow stream (Supplementary Information Movie 1), the cells could still be unevenly distributed vertically due to the inertial drift. While strategies such as viscoelasticity-based focusing are available to direct the cells toward the stagnation point of the cross-junction^50^, a viscoelastic medium may not be preferred for the extraction of mitochondria as the suspended medium may alter the functional properties of mitochondria^51^. However, given this suboptimal setting, the microscale cell shredding procedure has already shown improved performance compared to the two commercially available approaches. Particularly, it is observed that the efficiency of cell disruption drops consistently as the cell concentration decreased for Dounce Homogenizer and Qiagen Kit. In the case of Dounce Homogenizer, a highly likely scenario is that individual cells do not experience sufficient stress, either due to the lack of frictional forces from cell-cell interactions or the wall shear stress when very little of cells are introduced into the ~100 μm clearance gap between the cylinder and pestle. A similar rationale may apply to the Qiagen Kit, where a 1 mL syringe with needle (120 μm inner diameter) is adapted to disrupt the cell membrane. Cells may not be “shredded” sufficiently within the syringe needle. Furthermore, the buffer exchange steps in the Qiagen Kit also generate significant sample loss, rendering poor extraction efficiency at low concentrations. In the microscale cell shredder, the cells are stretched hydrodynamically on an individual basis regardless the concentrations of cells, and the membrane disruption efficiency is therefore outperformed the Dounce Homogenizer and Qiagen Kit at least across the concentrations range tested in this study. The optimal stress is able to deform the cell membrane sufficiently while the released mitochondria remain functional (as measured by mitotracker) and intact (as characterized by the amount of released citrate synthase). More importantly, the presented microscale cell shredder remains effective when handling samples volume of 200 μL containing around 10^4^ cells/mL.

## Conclusions

The lack of competent approaches for the extraction of subcellular organelles, such as mitochondria, from limited amount of samples has posed a remarkable barrier for the *in vitro* analysis of mitochondria from precious clinical samples. To this end, this study investigates the possibility of modulating hydrodynamic stress in a micro-channel of cross-junction to effectively disrupt the cellular membrane for the extraction of intact and functional mitochondria. The findings have attained a demarcated stress to shred the cellular membranes effectively, and yet keep the mitochondria unharmed, given that the elastic modulus of mitochondrial membrane is higher than that of the cellular membrane. The comparison with two commonly used approaches has demonstrated that the proposed “microscale cell shredder” yields at least 40% more functional mitochondria particularly at low cell concentrations (5–20 × 10^4^ cells/mL). We have shown the protocol may be faithfully optimized based upon the membrane stiffness by testing three model cell lines, namely human embryonic kidney cells, mouse muscle cells and neuroblastoma cells, as well as by hypertonicity altered membrane swelling. The presented microscale cell shredder is, to our knowledge, the first demonstration of extracting intact and functional mitochondria from mammalian cells *via* modulation of hydrodynamic stress. The ability to extract mitochondria from samples of clinically relevant size is expected to advance our understanding of mitochondrial dysfunction related disorders.

## Experimental methods

### Fabrication and operation of the microfluidic chip

The microchannel with a cross-sectional geometry of 30 μm in width and 60 μm in height was designed to produce an extensional stress at the range of 28–180 Pa given the volumetric flow rates in between 20–100 μL/min. The device was fabricated using standard soft lithography process^52^. Briefly, photoresist (SU8-2075) was spun on a 4” silicon wafer at the speed of 4000 r.p.m. to obtain a master mold of a height of 60 μm. The baking procedures were conducted following the manufacturer’s data sheet. Subsequently, polydimethylsiloxane (PDMS) base was well-mixed with the curing agent at 10:1 ratio. The mixture was then poured onto the SU8 master mold and cured at 60 °C for 1 h in an oven. The cured PDMS strip was peeled from the master, hole punched, and sealed with a cover glass gluing by a thin layer of PDMS. A syringe pump (Legato 100, KD Scientific or PHD 2000, Harvard Apparatus) was used to control the volumetric flow rate introduced into the chip.

### Cell culture

Human embryonic kidney cells (HEK293, ATCC, catalog no. CRL-1573) and mouse skeletal muscle cells (C2C12, ATCC, catalog no. CRL-1772) were maintained in Dulbecco’s Modified Eagle Medium (DMEM, Invitrogen, catalog no. 12100-046), supplemented with Fetal Bovine Serum (10%) (Invitrogen, catalog no. 10270-106), penicillin (100 units/mL) and streptomycin (100 μg/mL). Neuroblastoma cells (SH-SY5Y) was maintained in DMEM with nutrient mixture F-12, supplemented with Fetal Bovine Serum (10%), penicillin (100 units/mL) and streptomycin (100 µg/mL). All cells were grown at 37 °C in a humidified 5% CO_2_ atmosphere. Cells passages of 7 to 15 were used for HEK293 and C2C12, passages between 18–25 were used for SH-SY5Y cells.

### Cell disruption and mitochondria extraction

Cells were disrupted using three approaches, mechanical disruption (Dounce Homogenizer), the proposed microscale cell shredder and a commercially available chemical-based cell membrane disruption kit (Qproteome^®^ Mitochondria Isolation Kit), as detailed in the following. Dounce Homogenizer (Kimble® Tissue Grinder Comp, catalog no. 885300-0015) was utilized to mechanically disrupt cell membrane for mitochondria extraction. Cell disruption and mitochondria extraction were performed following a previously validated protocol^53^. Prior to mitochondrial extraction, the harvested cells were washed with pre-chilled phosphate-buffered saline (PBS, pH 7.4) and cell pellets were collected *via* trypsinization. Titrated concentrations (5–40 × 10^4^ cells/mL) of cells were swelled by resuspending in the hypotonic Reticulocyte Standard Buffer (RSB) (10 mM NaCl, 1.5 mM MgCl_2_, 10 mM Tris-HCl, pH 7.5) and incubated at 37 °C for 10 min. The swelled cells (200 µL) were then transferred into the cylinder of the homogenizer and optimized number of strokes were applied to produce cell lysates. The optimized number of strokes were chosen based on the maximal mitochondrial extraction and total protein yield as shown in Supplementary Figure S1. In the investigation for the effect of tonicity in cell disruption, cells were resuspended in the isotonic buffer (PBS, pH 7.4) and lysed subsequently. Mitochondria were then separated from the cell lysates by differential centrifugation^53^. Unbroken cell, nuclei, and cell debris were removed by centrifuging the lysates twice at 1,000 g for 5 min. The supernatant was then centrifuged at 15,000 *g* for 15 min to obtain the mitochondrial pellet. The pelleted mitochondria were suspended in 1× mitochondrial storage (MS) buffer (10 mM Tris-HCl, pH 6.7, 10 mM KCl, 0.15 mM MgCl_2_, 1 mM PMSF, and 1 mM DTT) and used directly for the subsequent characterizations.

For the microscale cell shredder, cells were swelled by incubating with the RSB hypotonic buffer (37 °C, 10 min), or resuspended in the isotonic buffer (PBS, pH 7.4) and shredded by flowing (200 µL) through the designed microfluidic chip at the optimized (80 μL/min) volumetric rate. Sample collected from the outlet was then processed following the differential centrifugation steps exactly as above-mentioned and the extracted mitochondria were stored in the 1× MS buffer and analyzed without further processing.

Qproteome^®^ Mitochondria Isolation Kit (Qiagen, catalog no. 37612) was used to chemically lyse the cells for mitochondrial extraction. The procedures for cell lysis and subsequent centrifugations were conducted following the manufacturer’s protocols. Firstly, fresh cell pellets were suspended in 200 µL of ice-cold Lysis Buffer (supplied by the Qiagen Kit), and incubated at 4 ˚C for 10 min. The cell solution was centrifuged at 1,000 g for 10 min. The precipitated cell pellets were then resuspended in 200 µL ice-cold Disruption Buffer (supplied by the Kit) and disrupted by repeatedly (10 times) passing through a narrow-gauge needle (22AWG, 120 µm inner diameter). Produced cell lysate was centrifuged at 1,000 g for 10 min to remove the large cell debris. The supernatant was then centrifuged at 6,000 g for 10 min to obtain the mitochondrial pallets. The mitochondrial pellets were washed with 200 µL Mitochondria Storage buffer (provided by the Kit) and finally isolated by centrifugation at 6,000 *g* for 20 min.

All the differential centrifugation steps were conducted at 4 °C using a high-speed refrigerated centrifuge (Neofuge 13 R, Heal Force).

### Flow cytometric analysis to determine cell disruption efficiency

The efficiency in cell disruption was determined using flow cytometer by analyzing the cell lysates immediately after disruption. Cell lysates were loaded into a 96-well plate (100 µL/well) and at least 10,000 events were counted by the Guava EasyCyte HT flow cytometer (Merck Millipore). Forward Scattering (FSC) and Side Scattering (SSC) signal intensities, correlated to the size and morphology of the cells, respectively, were plotted into the FSC-SSC plot for the identification of unbroken cells population in the total events of cell lysates^54^. Firstly, a control of FSC-SSC plot was profiled using a sample of intact cells and the total cell numbers (intact) were recorded. The cell lysates were then analyzed by the flow cytometer *via* the same settings and flow rate (0.59 µL/s). The FSC-SSC plots obtained from the lysates were compared with the control FSC-SSC plot to determine the fraction of disrupted cells or debris. The ratio between the counted events of disrupted cells and the events of total cells was defined as the percentage of cell disruption. The data were analyzed by the Guavasoft *v3.1.1* software.

### Quantification of functional mitochondria

The Mitotracker fluorescence dyes, capable of accumulating in the mitochondria based on mitochondrial membrane potential, was employed to quantify the amounts of functional mitochondria^43^. Extracted samples were stained with 2.5 µM Mitotracker Red (Thermo Fisher Scientific, catalog no. M22425) at 37 °C for 10 min and a relative amount of functional mitochondria was determined by flow cytometric analysis^55^. Mitotracker Red (excitation: 581 nm, emission: 644 nm) stained sample was loaded on a 96-well plate (100 µL/well) and 10,000 events were detected by the flow cytometer in the red channel (bandpass filter, 664 ± 10 nm). Red fluorescence positive events were determined by subtracting the population with background fluorescence intensity (where unstained mitochondria were used as the negative control^55^). The percentage of the Mitotracker red positive events was then multiplied by the number of events per unit volume to obtain the number of functional mitochondria (per unit volume).

### Bradford protein assay

Total protein concentrations of the extracted sample were determined by the Bradford protein assay^56^. Firstly, the Bradford reagent (Sigma Aldrich, catalog no. B6916) was brought to room temperature and 150 µL of reagent was added to 10 µL of extracted sample in a 96-well plate, incubated at 37 °C for 15 min. The absorbance was then measured at 595 nm using the SpectraMax (Molecular Devices) microplate reader. The measured absorbance was converted to the protein concentration by comparing with a standard curve constructed using the Bovine Serum Albumin (BSA) in the range of 10–50 µg/mL (Supplementary Figure S6a).

### Citrate synthase enzymatic activity of extracted mitochondria

The activity of citrate synthase (CS), specifically situating in the inner membrane of mitochondria, was utilized to differentiate the broken and intact mitochondria^44^, given that the enzyme would be released from the impaired or damaged mitochondria (due to the extraction procedures). Therefore, the activity of the released CS enzyme was measured as an exclusive marker to determine the mitochondrial membrane integrity^18,44^. All samples were processed following the manufacturer’s protocols (colorimetric CS assay kit, Sigma Aldrich, catalog no. MAK193). To start with, 50 µL of the reaction mixture (CS assay buffer, CS synthase developer and CS substrate mix) was added with 10 µL of extracted sample in a 96-well plate and incubated at 37°C. The absorbance at 412 nm was measured continuously until the absorbance reached a plateau (typically within 20–30 min) by a microplate reader (The SpectraMax). The CS enzymatic activity was then determined by comparing the measured optical density against a standard curve (Supplementary Figure S6b) constructed by Glutathione (GSH). Initial CS activity was determined by the absorbance measured immediately after adding the reaction mixture to the extracted sample. Subsequently, the CS Assay buffer (provided by the manufacturer) would gradually homogenize the mitochondria and eventually completely release the CS enzyme from the mitochondria. Upon the absorbance plateaued, the CS activity obtained then was defined as the “Total CS Activity”. The differences between the total CS activity and initial CS activity was referred to the “Latent Activity”. Finally, the membrane integrity of mitochondria in the sample was calculated by the equation below^18^:

$${{\rm{Mitochondrial}}\,{\rm{Integrity}}\left( {\mathrm{\% }} \right) = \frac{{{\rm{Latent}}\,{\rm{Activity}}}}{{{\rm{Total}}\,{\rm{Enzymatic}}\,{\rm{Activity}}}} \times 100}$$

Assay buffer and reaction mixtures have been equilibrated at room temperature before conducting the assay.

### Estimation of the mean shear stress and the extensional stress

The mean shear stress (*τ*) inside the microfluidic channel (at the cross-section of the necked channel) was estimated by the Newton’s law of viscosity as shown in Eq. (1)^57^:

1 $$\tau = \mu v\frac{{4y}}{{h^2}}$$

where *µ* is the fluid viscosity, *v* is the fluid velocity, *y* and *h* are the width and height of the channel, respectively. The extensional stress was estimated numerically using COMSOL Multiphysics^®^ *v5.1* (COMSOL Incorporation) for an incompressible Newtonian fluid under laminar flow conditions. Detailed simulation parameters are listed in Supplementary Text 1.

### High-speed imaging

The images were captured by a high-speed camera (Dimax CS3, PCO AG, Kelheim, Germany) in a frame rate ranged from 4,000 to 8,000 frames per second. The captured frames were processed with Advanced Research *v5.01* (NIS Elements, Nikon Incorporation, Japan) and retouched with Adobe Lightroom Classic CC *v7.0* (Adobe Systems).

### Statistical analysis

At least three independent experiments were analyzed for each data set. The results were plotted as mean ± SD. The statistical significance of all the analysis was determined by one-sided student’s *t* test with 95% confidence level.

## Electronic supplementary material

Supplementary Information Text and Figures

Supplementary Movie 1
