# Supplementary material for: Demarcating the membrane damage for the extraction of functional mitochondria
Source: Microsyst Nanoeng. 2018 Dec 31;4:39. doi: 10.1038/s41378-018-0037-y (PMC6311452; doi:10.1038/s41378-018-0037-y)
Supplement: Supplementary file 1 — Supplementary Information Text and Figures [file 41378_2018_37_MOESM1_ESM.docx]

Demarcating the Membrane Damage for the Extraction of Functional Mitochondria

*Md Habibur Rahman^1^, Qinru Xiao^1,2^, Shirui Zhao^1,2^, Fuyang Qu^1^, Chen Chang^3^, An-Chi Wei^3^,*

*Yi Ping Ho^1,2*^*

^1^Department of Biomedical Engineering, ^2^Shun Hing Institute of Advanced Engineering,

The Chinese University of Hong Kong, Shatin, New Territories, Hong Kong SAR, China

^3^Graduate Institute of Biomedical Electronics and Bioinformatics,

National Taiwan University, Taipei, Taiwan

*Correspondence: [*ypho@cuhk.edu.hk*](mailto:ypho@cuhk.edu.hk)*; Tel: +852 3943 434; Fax: +852 3942 1024*

**Supplementary Information**

| **Text 1:** | Detail information on simulation model to estimate the stress at the stagnation point of the cross-slot geometry. |
| --- | --- |
| **Figure S1:** | Optimization of the strokes number applied by the pestle of Dounce Homogenizer. |
| **Figure S2:** | Citrate synthase activity of the extracted mitochondria. |
| **Figure S3:** | Optimization of experimental conditions for neuroblastoma cells (SH-SY5Y). |
| **Figure S4:** | HEK293 cell disruption in isotonic (PBS) buffer. |
| **Figure S5:** | Cell deformation images. |
| **Figure S6:** | Bovine serum albumin (BSA) and glutathione (GSH) standard curves. |
| **Movie 1:** | Supplementary movie for the cells flow in the center streamline laterally. Cells were flowing at the volumetric flow rate of 20 µL/min and the movie was play backed at 10 fps. |

**Text 1:** Detail information on simulation model to estimate the stress at the stagnation point of the cross-slot geometry.

The shear stress that extended in the cross-slot geometry of the microfluidic channel was numerically estimated using commercially available Finite Element Analysis software COMSOL Multiphysics^®^. The parameters and definitions are given below:

Study: Laminar Flow, Incompressible Newtonian Fluid

Fluid Density: 1000 Kg/m^3^

Fluid Viscosity: 0.001 Pa.s

Channel Wall: No-slip boundary conditions.

Inlet: Fluid Velocity, *V* = Volumetric Flow Rate (*Q*)/Cross-section area (*A*)

Outlet: Pressure, *P =* 0

Mesh Definition: Physics-controlled Mesh.

Degree of Freedom: 145,456

Number of Elements: 152,289

**Figure T1a** denotes the boundary conditions applied in the 3D cross-slot microchannel and **Figure T1b** showed meshed elements near the cross-junction geometry. Extensional stress has been derivate from the relation between shear rate and dynamic viscosity of the fluid.


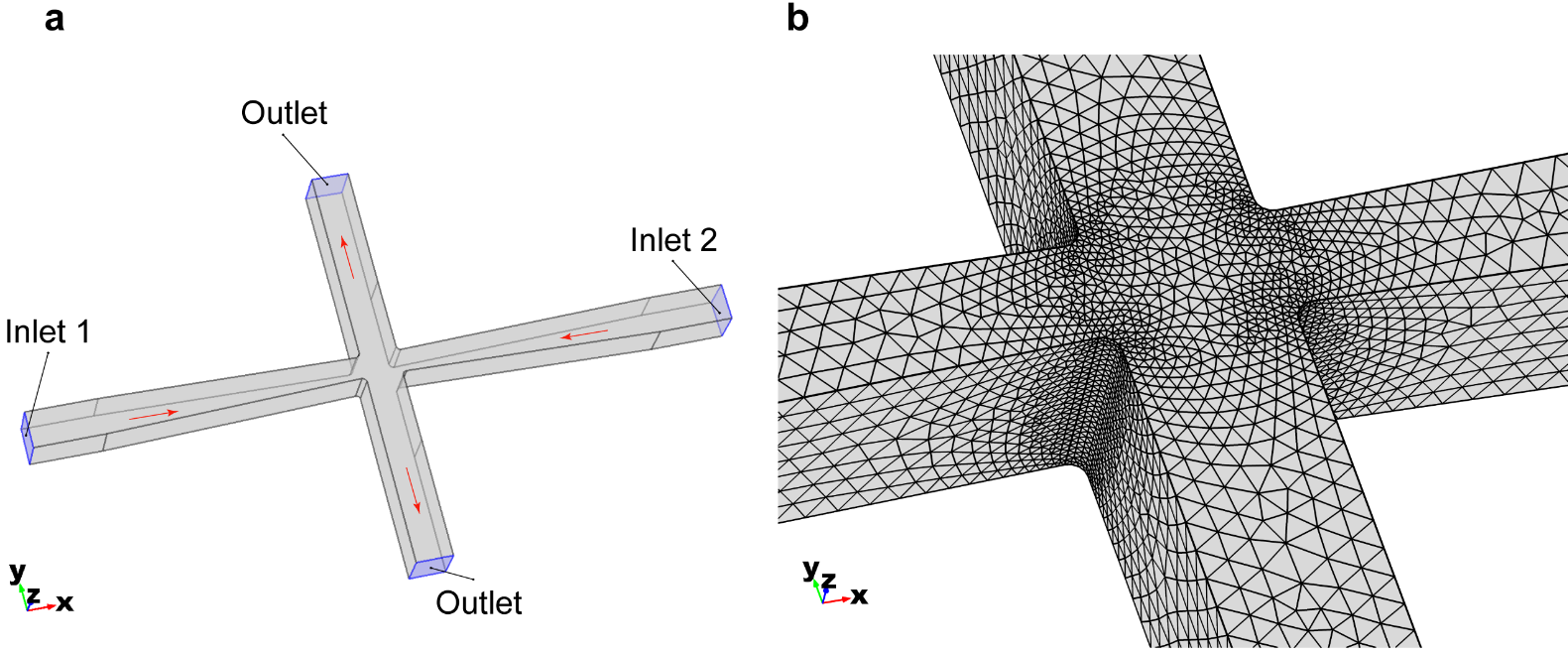


**Figure T1:** 3D geometry of the cross-slot microfluidics channel. **(a)** Overall geometry and the boundary conditions of the model. **(b)** Meshing of the elements as zoomed in the cross-slot region.


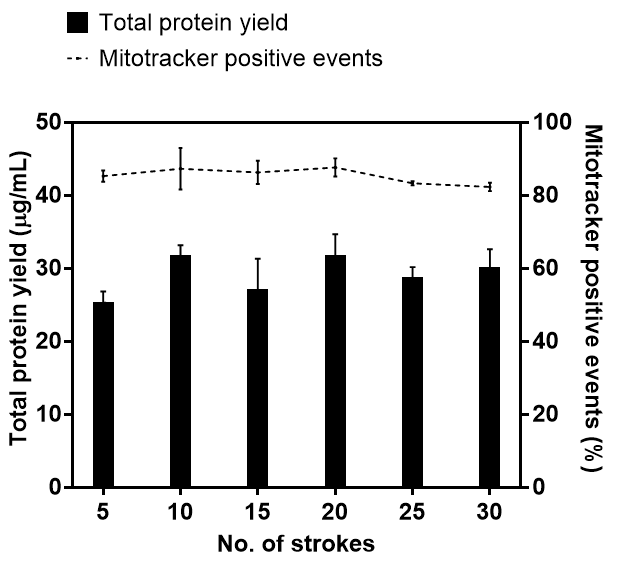


**Figure S1:** Optimization of the strokes number applied by the pestle of Dounce Homogenizer. Number of strokes applied by the homogenizer were optimized by the maximum percentage of mitotracker positive events and the plateaued value of the protein yield. Results were obtained by disrupting HEK 293 cell lines (10^6^ cells/mL) and plotted as mean ± SD (n=3 independent experiments).


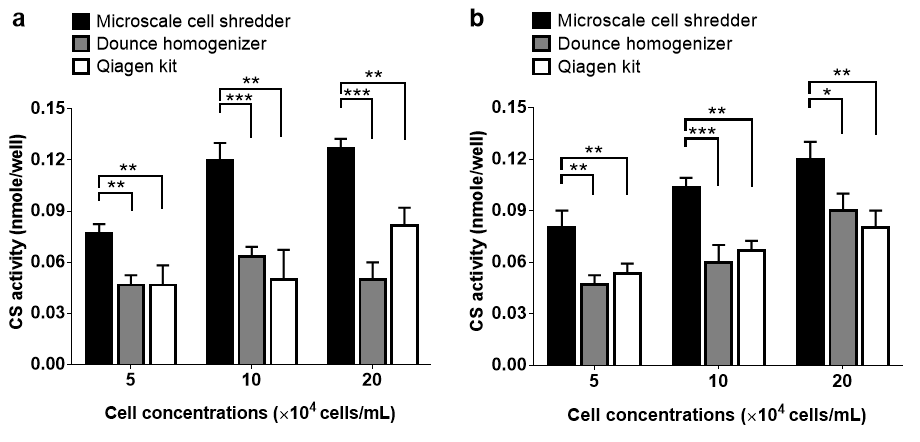


**Figure S2:** Citrate synthase activity of the extracted mitochondria. Total citrate synthase activity measured from the extracted sample using **(a)** HEK293 cells, and **(b)** C2C12 cells. Results were plotted as mean ± SD (n=3 independent experiments, *P<0.05, **P<0.01, ***P<0.001).


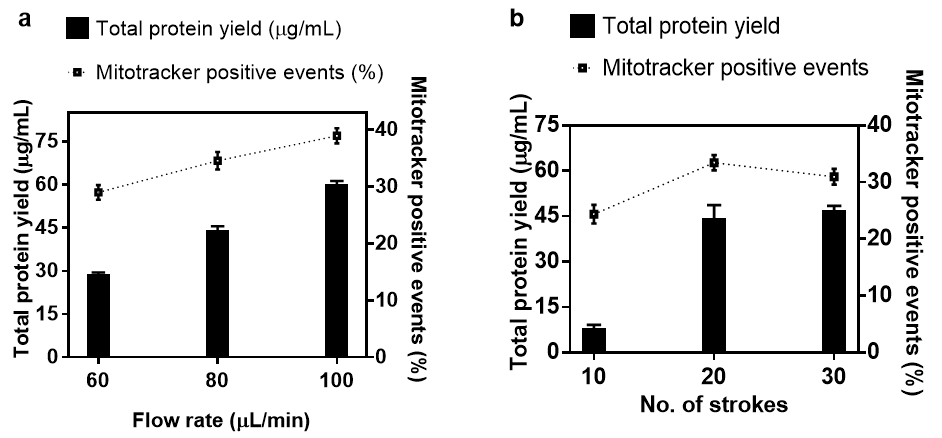


**Figure S3:** Optimization of experimental conditions for neuroblastoma cells (SH-SY5Y). **(a)** Flow rates for microscale cell shredder, and **(b)** number of strokes for Dounce Homogenizer were optimized based on total protein yield and mitotracker red positive percentage. Experiments were conducted at the cell concentrations of 10^6^ cells/mL and results were plotted as mean ± SD (n=3 independent experiments).


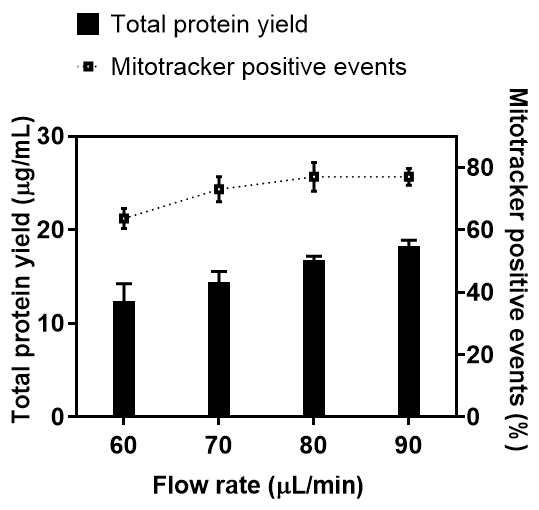


**Figure S4:** HEK293 cell Disruption in isotonic (PBS) buffer. Experiments were conducted at the cell concentrations of 10^6^ cells/mL. The total protein yield and mitotracker red positive signal results were plotted as mean ± SD (n=3 independent experiments).


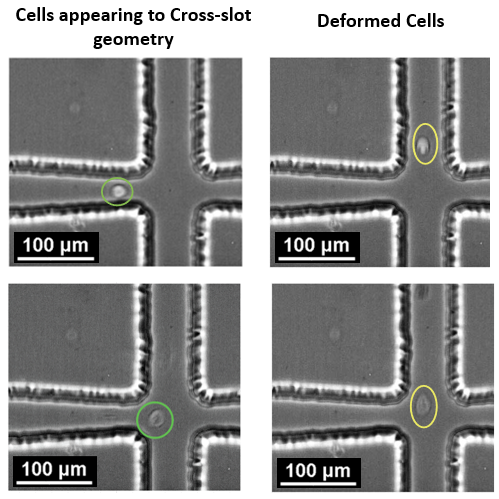


**Figure S5:** Cell deformation. Images were recorded by flowing HEK293 of a concentration 10^6^ cells/mL at the flow rate of 40 µL/min.

P


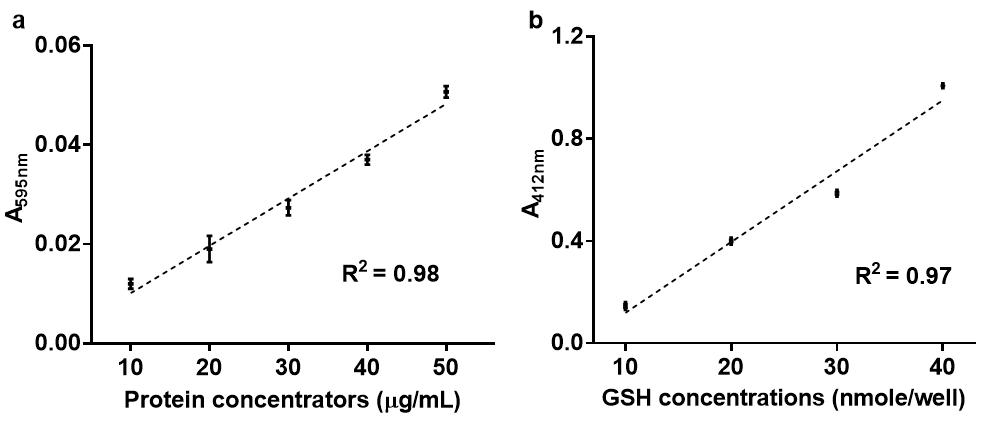


**Figure S6:** Standard curves. **(a)** Bovine Serum Albumin (BSA) protein standard curve was constructed by the Bradford assay. **(b)** Glutathione (GSH) standard curve to determine CS activity (reagents supplied by the manufacturer of the CS Assay Kit). Curves were fitted with linear regression at 95% confidence intervals.
